# The Role of G Protein-Coupled Receptor Kinase 6 Regulation in Inflammation and Pain

**DOI:** 10.3390/ijms232415880

**Published:** 2022-12-14

**Authors:** Maike Stegen, Ulrich H. Frey

**Affiliations:** 1 Department of Anaesthesiology and Intensive Care Medicine, Essen University Hospital, University of Duisburg-Essen, Hufelandstr. 55, D-45147 Essen, Germany; 2 Department of Anaesthesiology, Operative Intensive Care Medicine, Pain and Palliative Medicine, Marien Hospital Herne, Ruhr-University Bochum, Hölkeskampring 40, D-44625 Herne, Germany

**Keywords:** GRK6, inflammation, pain, receptor regulation

## Abstract

The G protein-coupled receptor kinase 6 is associated with inflammation and pathological pain. Impairment of GRK6 expression was described in chronic inflammatory diseases such as rheumatoid arthritis and this was shown to be accompanied by an imbalance of downstream signaling pathways. Here, we discuss novel aspects of GRK6 interaction and its impact upon hyperalgesia and inflammatory processes. In this review, we compile important findings concerning GRK6 regulation for a better pathophysiological understanding of the intracellular interaction in the context of inflammation and show clinical implications—for example, the identification of possible therapy goals in the treatment of chronic inflammatory hyperalgesia.

## 1. G Protein-Coupled Receptor Kinases

G protein-coupled receptor kinase 6 (GRK6) is part of the family of G protein-coupled receptor kinases (GRK), which expands over hitherto seven identified serine/threonine kinases. Owing to sequence homologies, they are divided into three subfamilies [1]. GRK1 and -7 are known as visual kinases as they are predominately expressed in retinal rods and cones [2,3]. GRK2 and -3 are expressed ubiquitously and represent the β-adrenergic receptor kinase family. GRK4, -5, and -6 belong to the GRK4 subfamily [4]. GRK5 and -6 are expressed in a broad variety of tissues, with a specifically enriched expression of GRK6 in plasma cells and lymphoid tissue, whereas GRK4 expression is limited to testicular, cerebellar, and renal cells [5,6,7,8].

The initially described main process regulated by GRKs is the phosphorylation of agonist-bound G protein-coupled receptors (GPCR), leading to receptor downregulation—a process called homologous receptor desensitization [9,10]. As of today, GPCRs form the largest group of receptors known in eukaryotic cells [11]. They are characterized by a hepta-helical structure encompassing seven transmembrane receptor loops and are coupled to small hetero-trimeric G proteins [12]. Owing to the subtype of the coupled Gα subunit, there are several possible signaling pathways induced by the receptor [12]. Their signaling is crucial for most physiologic processes, including autonomous nervous system regulation [13], sensory perception [14,15], nociception [16], and inflammation [17]. In addition to the well-known classical GPCR signaling pathways, there is a plethora of other possible interactions and downstream pathways induced by GPCRs. For instance, GPCRs can exist in a constitutively active conformation [18], initiate signaling via the Gβ/γ subunits [19] and induce signaling cascades independent from G proteins [20].

**Figure 1:**
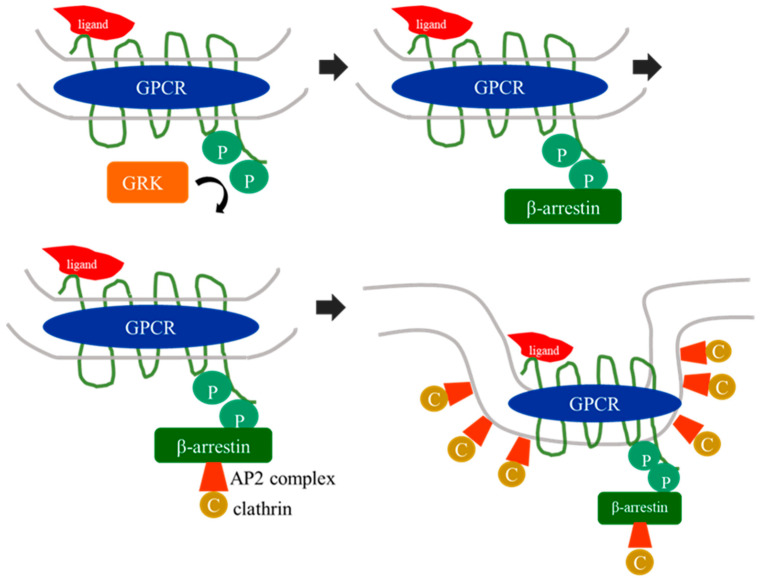
Concept of homologous receptor desensitization (simplified chart). GRK-mediated phosphorylation of a ligand-bound GPCR leads to recruitment of β-arrestin, which enables accessory protein 2 (AP2) and clathrin binding. Consequently, clathrin-coated pits are set up and the receptor is internalized by dynamin-mediated formation of an endocytic vesicle. P: phosphorylated residues. C: clathrin [22,23].

GRK-induced phosphorylation of GPCRs classically takes place at serine and threonine residues at the carboxy-terminal or third cytoplasmatic receptor loop [9]. Subsequently, proteins of the β-arrestin family are able to bind to the phosphorylated residues and establish a steric hindrance of G protein binding to the receptor finally leading to signal shutdown (Figure 1) [21,22]. Furthermore, a successive endocytic internalization process of the GPCR is initiated. Corresponding to specific ubiquitination patterns of the bound β-arrestins, the receptor is the target of lysosomal degradation, or can be subjected to dephosphorylation and reintegration in the cell membrane as an active receptor [9,21].

**Figure 2:**
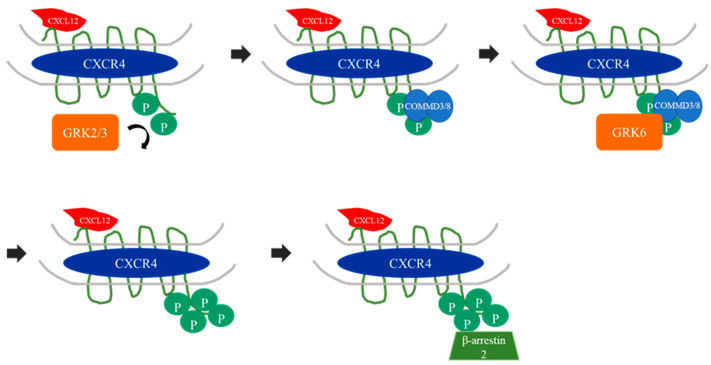
Possible mechanisms of COMMD3/8-mediated targeting of GRK6 to CXCR4 (simplified chart). Phosphorylation of the carboxy-terminal serine-353 and -354 by GRK2 or GRK3 seems to be a prerequisite for COMMD3/8-binding to CXCR4. In the following, GRK6 is recruited to the receptor site and conducts further phosphorylation, culminating in a binding of β-arrestin 2 and presumably initiating MAP-kinase-related signaling pathways [25].

Nonetheless, GRK signaling expands to a much broader spectrum of possible interactions. Distinct phosphorylation patterns at the receptor can initiate binding of different arrestin subtypes. In this concept of receptor “barcoding”, the successive conformation of the receptor–arrestin complex, imposed by differentiated phosphorylation patterns, specifies which downstream signaling cascade is initiated [24]. Recent studies identified a complex of copper metabolism MURR1 domain-containing proteins (COMMD) 3 and 8, which binds to the carboxy-terminal region of CXC-receptor 4 (CXCR4) and is responsible for recruitment of GRK6 to the receptor [25]. Therefore, it represents an important connecting link between receptor phosphorylation and arrestin binding [25]. In the case of CXCR4 phosphorylation by GRK6, the COMMD3/8 complex paves the way for β-arrestin 2 recruitment to the receptor, and subsequently promotes activation of mitogen-activated protein (MAP)-kinase signaling (Figure 2) [25].

## 2. Characteristics of G Protein-Coupled Receptor Kinase 6

**Figure 3:**
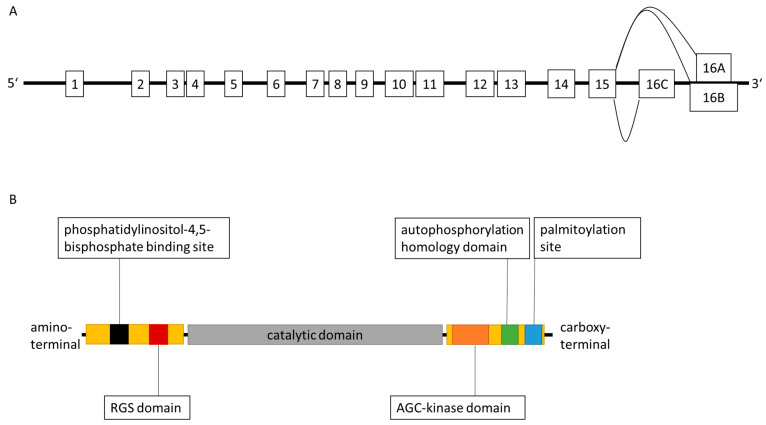
(**A**) Schematic overview of GRK6 gene structure and carboxy-terminal region with alternative splicing of variants GRK6A, B and C. The boxes depict exons. Simplified chart [4]. (**B**) Schematic overview of GRK6 protein structure, simplified chart [29]. The amino-terminal domain contains a phosphatidylinositol-4,5-bisphosphat binding site as well as a regulator of G protein signaling binding domain (RGS domain). This domain is also used for phosphorylation of G protein-coupled receptors and inhibition of receptor signaling [40]. The carboxy-terminal region contains an AGC-kinase domain, which is highly conserved in several serine-threonine kinases [41]. GRK6 also contains an autophosphorylation homology domain similar to GRK5, albeit, even in presence of phospholipids autophosphorylation was not detected in GRK6 proteins [42]. Finally, the GRK6A splice variant undergoes palmitoylation at the carboxy-terminus [30,31].

The GRK6 gene (Entrez gene code 2870) is located on chromosome 5q25 and has a transcriptionally inactive pseudogene on chromosome 13 [26,27]. It has 16 exons and 15 introns and its protein has a mean molecular weight of 66 kDa [4,28]. G protein-coupled receptor kinases share the structural characteristic of a central catalytic domain, which is highly conserved within the different members of the GRK family [29]. The amino-terminal region serves as a binding link to the GPCR and may also bind to phosphatidylinositol-4,5-bisphosphate, whereas the GRK’s ability for membrane binding is attributed to the carboxy-terminal region, for it is subjected to post-translational modifications (Figure 3) [29]. The GRK6 carboxy-terminal region exists in a palmitoylated form and its palmitoylation, as well as a C-terminal amphipathic helix motif, contribute to the attachment to the cell membrane [30,31]. Three GRK6 splice variants were described—GRK6A, B, and C, with a length of 576, 589 and 560 amino acids, respectively (Figure 3) [4,32]. GRK6B and C lack the carboxy-terminal palmitoylation site and therefore less enzyme activity or membrane attachment in comparison to GRK6A was suggested. Interestingly, all three splice variants were shown to attach strongly to the cell membrane [31]. In addition, the GRK6B splice variant contains a protein kinase A consensus binding site, which is similar to the GRK5 membrane localization site [4].

In addition, GRK6 can be found in the nuclear subcellular fraction due to a nuclear localization sequence (NLS), which is located at the amino acids 388 to 395 and is similar to the NLS in GRK5 [33,34]. A potential role of both GRKs in the regulation of DNA transcription therefore is conceivable. Strikingly, GRK5 and 6 are able to directly bind DNA via their NLS [33,34]. Furthermore, GRK6 was described to interact with transcription factors, such as the downstream regulatory element antagonist modulator (DREAM) [35]. Mainly, non-palmitoylated GRK6 proteins were detected in the nuclear fraction, and de-palmitoylation seems to be a prerequisite for GRK6 proteins to shuttle in and out of the nucleus [32].

As of today, few mechanisms of the transcriptional regulation of GRK6 expression are known. GRK6 transcription is positively regulated by the CCAAT-enhancer binding protein α (C/EBPα), binding to hitherto three identified binding sites in the GRK6 promoter [36]. In addition, a cAMP responsive element binding protein (CREB)—binding site at position-356 to -348 in relation to the translation starting point was disclosed [37]. With regard to epigenetic modifications of the GRK6 regulation, multiple investigations concerning the methylation status were performed, in particular in the context of malign tumour diseases. GRK6 was found to be hypermethylated in the adenocarcinoma of the lung, and a positive methylation status, resulting in decreased GRK6 expression levels, was associated with poor survival rates [36,38]. Comparable results were achieved by investigating the methylation patterns of the GRK6 promoter in hypopharynx squamous cell carcinoma [39].

## 3. GRK6 in the Clinical Setting of Inflammation and Inflammatory Hyperalgesia

Several studies revealed an association of GRK6 with chronic inflammatory diseases. In patients suffering from rheumatoid arthritis, an overall decrease in GRK activity could be detected in peripheral blood mononuclear cells [43]. Concomitantly, cAMP levels were increased in these patients, suggesting an enhanced activity of the adenylate cyclase, which might be mediated by less downregulation of Gs-coupled receptors due to lack of G protein-coupled receptor kinases [43]. More precisely, cytosolic and membrane-bound GRK6 and GRK2 protein expression levels were decreased, which was reproduced in the latter case by cell treatment with interferon-γ and interleukin-6 (IL-6) [43]. Induced arthritis in rats led to a comparable decrease in GRK2 and GRK6 protein expression levels in splenocytes as well as in activated mesenterial lymph node cells [44]. Notably, these reductions were dominant on protein level, but not reflected on mRNA level, suggesting that post-transcriptional or even post-translational processes are responsible for decreased protein expression [44]. A similar decrease in cytosolic GRK activity was observed in Agouti rat splenocytes in a model of induced encephalomyelitis, which was accompanied by a significant decrease in GRK2 and GRK6 expression levels [45]. In addition, patients who underwent cardiopulmonary bypass surgery showed a post-operative reduction in GRK6 expression in peripheral blood mononuclear cells [46]. Cardiopulmonary bypass surgery results in a systemic pro-inflammatory response, initially driven by interleukin-6, which parallels the inflammatory situation in chronic diseases such as rheumatoid arthritis, and might represent a direct causal connection to the detected decrease in GRK6 expression [46].

Taken together, these findings suggest that GRK6 is decreased in secondary lymphatic organs and peripheral blood lymphocytes in the context of acute inflammatory situations. This decrease mainly seems to underlie a post-transcriptional regulatory mechanism and was propagated by pro-inflammatory cytokines, for instance by interleukin-6 and interferon-γ.

Notably, in most investigations described here, GRK6 seems to act as a GRK2 supporting enzyme, whose overall enzyme activity was more dominant in immune organs in comparison to GRK6, and whose decrease in expression upon inflammatory stimuli was principally more profound. This could give rise to the assumption, that GRK6 might play a minor role not only in the inflammatory, but also in the entire cellular regulation in comparison to GRK2. In accordance with this assumption, GRK2 knockout mice are not viable, whereas knockout of GRK6 expression does not impair overall survival. Consequently, a multitude of GRK6 knockout investigations were performed, and several thereof in the context of inflammation.

## 4. GRK6-Knockout Studies Addressing Inflammation and Hyperalgesia

GRK6-knockout studies demonstrated that GRK6 deficiency is associated with increased sensitivity for pain, also known as hyperalgesia. Firstly, signs of increased post-inflammatory visceral hyperalgesia in response to intracolonic treatment with capsaicin could be detected in female GRK6-knockout mice in comparison to wild-type mice after recovery from dextran sodium sulphate triggered colitis [47]. This hyperalgesia was not accompanied by altered colon histology, increased production of the cytokines TNF-α, IL-1β and IL-10 in GRK6-deficient mice, nor was there any evidence of increased granulocyte stimulation [47].

In a similar model of inflammatory bowel disease in mice, GRK6-deficient granulocytes showed an increased chemotactic response to the pronounced pro-inflammatory colon milieu in acute inflammation [48]. This was accompanied by a higher degree of acute severity of symptoms, of colon length shortening, and of keratinocyte-derived chemokine distribution in knockout animals compared to wild-type littermates [48]. Additionally, acute dextran sodium sulphate induced colitis showed a higher rate of transition to chronic colitis in GRK6-deficient mice [48]. The same group showed that post-inflammatory pain sensitivity was also increased in GRK6-knockout mice after stimulation with TNF-α and IL-1β, and this was not only evident in a somatic, but also in a neuropathic pain model in response to mechanical and thermal stimuli [49]. In addition, IL-1β and TNF-α stimulation in GRK6-deficient mice resulted in increased signaling via the p38 pathway, whereas baseline concentrations of phosphorylated p38 were not altered in GRK6-knockout mice in comparison to wild-type animals [49]. Furthermore, PI3-kinase/Akt-signaling was decreased in GRK6-deficient mice after IL1-β and TNF-α stimulation, suggesting that this pathway is diminished in the context of GRK6 deficiency in favor of expanded signaling via p38 [49]. 

In a bacterial lung infection model in GRK6-deficient mice, delayed apoptosis of neutrophil granulocytes and an accumulation of neutrophils in bronchoalveolar lavage specimen was detected due to an impaired transendothelial and transepithelial neutrophil migration [50].

Furthermore, GRK6-knockout mice were more susceptible to K/BxN serum transfer-induced arthritis than wild-type littermates, which came along with elevated IL-6 serum levels [51].

In accordance with these findings, chronic nerve constriction injury in rat dorsal root ganglia led to a significant downregulation of GRK6, accompanied by thermal hyperalgesia and mechanical allodynia [52]. Conversely, GRK6 overexpression resulted in alleviation of pain responses and suppression of CXCR2 signaling [52].

However, another model of spinal cord injury in rats found an upregulation of GRK6 expression, particularly in microglia, giving evidence for a neuroinflammatory-related response [53]. This upregulation was also reproducible upon lipopolysaccharide stimulation in an immortalized rat microglial cell line [53].

## 5. GRK6 in Autoimmunity

GRK6-deficient mice developed an autoimmune syndrome, which was comparable to systemic lupus erythematosus [5]. This autoimmune reaction was probably based on the phenomenon of impaired phagocytic abilities of GRK6-deficient phagocytes, especially of macrophages, leading to a disturbed clearance of apoptotic material. This was particularly evident in splenic tissues of GRK6-deficient mice, where senescent red blood cells, as well as B cells, were removed less efficiently [54]. Under physiological conditions, apoptotic cell material is immediately and carefully taken up by local phagocytes [54]. The accumulation of apoptotic cells, however, and their decay in a process of secondary necrosis with succeeding release of intracellular metabolites, such as ATP, initiates and maintains an inflammatory response. This may contribute consequently to a false identification of the normally compartmentalized and internalized intracellular material as “non-self” [55]. Henceforth, it may cause auto-inflammatory syndromes such as systemic lupus erythematosus [54]. To date, GRK6 is known to interact with GIT1 to induce uptake of apoptotic material; however, it does not appear to utilize conventional pathways such as Rac1/PAK/MEK/ERK1/ERK2, GULP/Rac1 or DOCK180/ELMO/Rac1 [54]. Further studies need to uncover the exact mechanism GRK6 holds to contribute to uptake of apoptotic material.

## 6. The Potential Role of GRK6 in Chemotaxis

Chemokines derived from chemotactic cytokines are important mediators in inflammation. They are produced by leukocytes, but also by various other cell types, mainly upon inflammatory stimulation, and usually bind to G protein-coupled receptors [56,57]. Nevertheless, a plethora of other downstream signaling pathways upon chemokine receptor activation are known, including JAK/STAT or mitogen-activated protein (MAPK)-signaling [58]. The initially described process directed by chemokines in inflammation is the contribution to leukocyte extravasation by induction of integrin formation and chemotaxis in inflammatory tissues [56]. Furthermore, homeostatic chemokines regulate the maturation of leukocytes in hematopoiesis and of the adaptive immune system in thymus gland and lymph nodes [59]. The CXC chemokine group comprises 16 members [57]. GRKs seem to play a pivotal role in the regulation of chemokine receptor signaling. In particular, GRK2, 3, 5 and 6 are involved in chemokine receptor regulation [60]. Besides the suppression of CXCR2 signaling through GRK6 upregulation as mentioned before [52], multiple studies could show an association of GRK6 expression with CXCR4 signal transduction.

The CXCR4 is uniquely bound by CXC-ligand (CXCL) 12, also known as stromal cell-derived factor 1α (SDF1α), and is crucial for the cardiovascular, hematopoietic and central nervous development in embryogenesis (Table 1) [61]. In inflammation, it is an important regulator of neutrophil chemoattraction and lymphocyte extravasation [58]. Some chronic inflammatory diseases are associated with elevated CXCL12 plasma concentrations, among them rheumatoid arthritis and chronic inflammatory bowel diseases [62,63].

CXCR4 downregulation and degradation via ubiquitination upon CXCL12 binding are mediated by GRK6 phosphorylation of receptor serine residues [64,65]. Additionally, GRK2 and 3 are involved in CXCR4 receptor regulation, composing a barcode pattern at the C-terminal receptor leading to the activation of distinct intracellular signaling pathways [60]. GRK6 activated recruitment of β-arrestin 2 to CXCR4 and finally resulted in ERK1/2 signaling in HEK293 cells (Figure 2) [25,65]. In HeLa cells, GRK6 depletion resulted in decreased rates of CXCR4 downregulation, and less active ERK1/2 signaling [66]. Studies in GRK6-deficient mice showed, that chemotactic abilities and transendothelial migration capacities of predominately T lymphocytes and, to a lesser extent, B lymphocytes were impaired upon CXCL12 stimulation in comparison to wild-type littermates [67]. Simultaneously, β-arrestin 2-deficient mice showed the same deficiency in T and, of secondary importance, in B-cell diapedesis and chemotaxis [67]. This suggests that GRK6 interaction with CXCR4 not only encompasses its downregulation but is also necessary for efficient signaling and cellular function. Therefore, a GRK6- and β-arrestin 2-mediated pathway may be a prerequisite for orchestration of T and B lymphocyte chemotaxis. In contrast, a study of bone marrow-derived polymorphonuclear neutrophil chemotaxis in response to CXCL12 stimulation showed an enhanced approach in GRK6-deficient mice in comparison to wild-type mice [68]. Obviously, CXCR4-mediated cell reaction in GRK6 deficiency might show cell type-specific differences. These findings were associated with a reduced rate of CXCR4 desensitization and impaired mobilization of bone marrow neutrophils into the peripheral circulation in response to G-CSF stimulation in GRK6-deficient mice [68]. CXCR4 signaling in conjunction with GRK6-mediated pathways might therefore be crucial for neutrophil mobilization and GRK6 deficiency may lead to an impaired response of the innate immune system, either by reduced CXCR4 downregulation, or by deficiency of another activating CXCR4-GRK6-mediated pathway, which has not yet been explained in detail.

In addition to the overshooting neutrophil response to CXCL12 in GRK6 deficiency, GRK6-deficient granulocytes were also more susceptible to leukotriene B4- and C5a anaphylatoxin-promoted chemotaxis than wild-type granulocytes [51]. As a member of the eicosanoid family, leukotriene B4 is a key regulator of inflammatory responses in particular in granulocytes, macrophages, and mast cells [69]. It has a distinct chemotactic potential and induces the production of further pro-inflammatory cytokines [69]. Disturbed leukotriene B4 homeostasis is associated with chronic inflammatory diseases. For instance, leukotriene B4 was shown to contribute to inflammatory arthritis [70]. GRK6 was able to efficiently desensitize leukotriene B4 receptor 1 (BLT1) [71], which gives rise to the assumption that GRK6 deficiency may maintain overshooting leukotriene B4-mediated processes under inflammatory circumstances (Table 1). Indeed, in the absence of GRK6, chemotactic activity of neutrophils was significantly increased in response to leukotriene B4 stimulation and clinical inflammatory reaction to arachidonic acid-infiltration was fortified in GRK6-deficient mice [72].

GRK6 is also known to interact with chemokine receptor CCR7 (Table 1) [73]. CCR7 is highly expressed in T cells and mediates cell circulation in secondary lymphoid tissues. This receptor is bound by chemokines CCL19 and CCL21 [73]. In this context, GRK6 seems to contribute to β-arrestin recruitment and activation of MAP-kinase signaling in response to both receptor ligands [73]. CCR7 is also regulated by other GRK subtypes. For example, GRK3 is responsible for inducing CCL19-dependent haptotaxis in dendritic cells [60].

Moreover, GRK6 is proposed to regulate chemokine-like receptor 1 (CMKLR1)-signaling, which is bound by chemerins leading to a pro-inflammatory cellular response; or alternatively bound by resolvin E1, resulting in an anti-inflammatory response (Table 1) [74]. Involvement of chemerin is, for instance, described in the pathophysiology of rheumatoid arthritis and lupus nephritis [75,76]. GRK6 was shown to induce β-arrestin 2 coupling to chemerin-bound CMKLR1 receptors and seems to contribute to CMKLR1 internalization under physiologic conditions [74]. Therefore, CMKLR1 desensitization might be mediated via a β-arrestin 2- and clathrin-dependent pathway. Furthermore, GRK6- and β-arrestin 2-deficient macrophages showed increased migration upon stimulation with chemerin, which was incidental to increased Akt phosphorylation in GRK6-deficient cells [74].

GRK6 is also involved in the downregulation of calcitonin gene-related peptide (CGRP) receptors (Table 1) [77]. CGRP is a neuropeptide positively associated with the sensation of pain [78]. For instance, CGRP levels in local tissue as well as in peripheral blood cells were reported to be elevated after burn injuries, concomitantly to a local inflammatory response with increased levels of TNF-α [79]. GRK6 deficiency may therefore lead to a misbalance in CGRP receptor signaling due to impaired signal shutdown, and consequently, may contribute to hyperalgesia.

**Table 1:** GPCRs and their interaction with GRK6.

Receptor	Main G Protein Coupled	Main Ligand	Main Signaling Pathways Induced	Impact of GRK6
calcitonin gene-related peptide receptor (CGRP-R)	G_s_ G_i/o_ G_aq/11_	calcitonin gene-related peptide (CGRP)	- activation of cAMP production via adenylate cyclase - calcium mobilisation via phospholipase C β [80]	GRK6 is involved in receptor downregulation [79]
C-C receptor 7 (CCR7)	G_i_	CCL19 > CCL21	- inhibition of cAMP production - β arrestin- mediated MAP kinase signaling [81]	GRK6 deficiency impairs β arrestin recruitment to CCR7 [73]
chemokine-like receptor 1 (CMKLR1)	G_i_ G_o_	chemerin resolvin E1	- calcium mobilisation via phospholipase C β - inhibition of cAMP production - MAP kinase signaling - phosphatidylinositol 3- kinase (PI3K) signaling [82]	GRK6- and β arrestin 2-deficiency leads to increased migration upon chemerin binding [74] GRK6 deficiency leads to increased Akt phosphorylation upon chemerin binding [74]
C-X-C receptor type 4 (CXCR4)	G_i_	CXCL12 (also titled SDF1α)	- extracellular signal-related kinases (ERK) 1/2	mutation of CXCR4 leads to impaired receptor silencing inter alia by GRK6 and results in WHIM syndrome (papilloma-virus induced warts, hypogammaglobulinemia, bacterial infection, myelokathexis) [66] GRK6-deficiency leads to impaired chemotaxis and transepithelial migration in B and T lymphocytes [67] GRK6-deficient neutrophils showed enhanced chemotaxis but reduced mobilisation from the bone marrow to the peripheral blood circulation [68]
leukotriene B4 receptor 1 (BLT1)	G_i_ [83]	leukotriene B4	- activation of phospholipase C β via the Gβγ subunit, Ras, PI3K [84]	GRK6 deficiency promotes increased chemotactic activity in neutrophils and leads to an increased inflammatory reaction in response to arachidonic acid [72]
platelet- derived growth factor β receptor (PDGF-R)	tyrosine kinase receptor	platelet-derived growth factor	- MAP kinase signaling [85] - phospholipase C γ [85] - phosphatidyl- inositol 3-kinase [85]	activation of PDGF-R is associated with decreased GRK6 expression [86]

## 7. GRK6 in the Context of Transcriptional Regulation Mediated by NFκB

GRK6 might play a pivotal role in the regulation of nuclear factor κ B (NFκB) in the context of inflammation. NFκB is an ubiquitously expressed transcription factor and regulates a plethora of cellular processes. Importantly, it is a key regulator of inflammatory gene transcription and ensures, among other things, the expression of inducible NO synthase (iNOS), interleukin 1-β, interleukin-6, interleukin-8, TNF-α, and interferon-γ [87]. Therefore, the investigation of NFκB regulation is of tremendous importance for the understanding of inflammatory cell processes.

**Figure 4:**
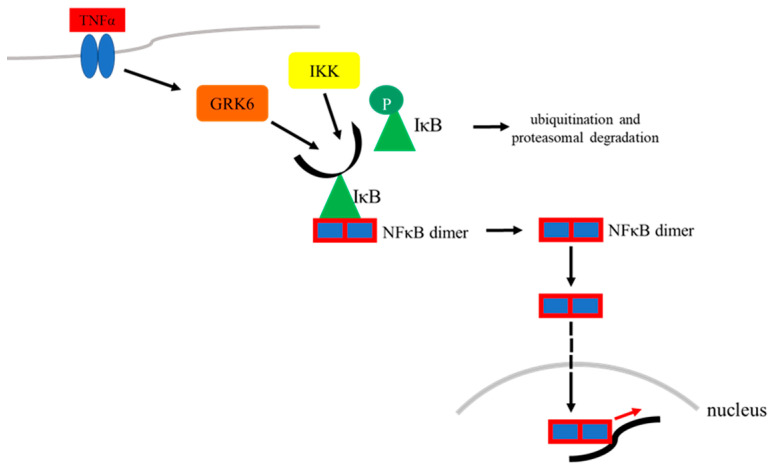
Influence of TNF-α induced activation of GRK6 on NFκB signaling. GRK6 corresponds to the inhibitor of NFκB kinase (IKK) in its phosphorylating function to the inhibitor of NFκB (IκB) [88]. Therefore, both enzymes initiate IκB’s ubiquitination and proteasomal degradation. The unbound NFκB dimer subsequently translocates to the nucleus and induces gene transcription.

Studies demonstrated that GRK6 is involved in the TNF-α induced inflammatory signal cascade by interacting with the inhibitor of nuclear factor K B (IκB) [88]. Strikingly, GRK6 directly phosphorylates IκB at serine 32 and 36, which finally results in ubiquitination and proteasomal degradation of IκB (Figure 4) [88]. As a result, the NFκB dimer is released and translocates to the nucleus, where it takes part in pro-inflammatory gene transcription (Figure 4). Conversely, GRK6 knockdown suppressed the otherwise TNF-α-induced transcription of IL-1β, IL-6 and iNOS via NFκB [88].

Neonatal maternal deprivation led to specifically reduced GRK6 expression levels in the arcuate nucleus of rats, which was also accompanied by chronic visceral pain in a model of chronic inflammatory bowel disease [89]. Notably, GRK6 overexpression mitigated chronic visceral pain in these rats [89]. GRK6 downregulation in the arcuate nucleus was associated with a cell type-specific reduced expression of NFκB, which gives insight into a possible regulation of NFκB expression by GRK6 [89]. This suggests that GRK6 not only could be an activator of NFκB-related gene transcription but may also be a promotor of NFκB expression itself.

Both hypotheses are further supported by findings of Duan et al. showing a correlation of GRK6 upregulation and presumably NFκB-mediated expression of pro-inflammatory cytokines, such as IL-8, in a chondrocyte model of osteoarthritis upon stimulation with IL-1β [90].

## 8. GRK6 Contributes to Reduced Hypoxia-Induced Factor 1-α Activity

**Figure 5:**
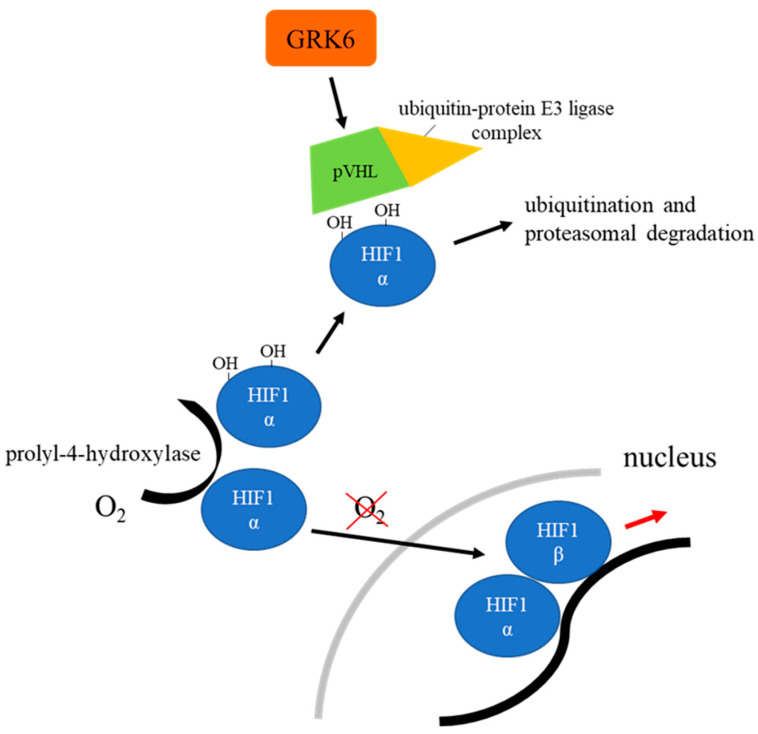
Regulatory cascade of HIF1α (simplified chart). In normoxia, hydroxylated HIF1α is bound by pVHL, which initiates its downregulation. GRK6 seems to have a stabilizing effect on pVHL. Under hypoxic conditions, however, HIF1α dimerizes with HIF1β and induces gene transcription [91,92].

In alveolar epithelial cell type II, inhibition of GRK6 led to an increase in hypoxia-induced factor 1-α (HIF1α) expression and activity [91]. In the adenocarcinoma of the lung, GRK6 expression is known to be suppressed, which is associated by elevated levels of HIF1α protein expression [91]. This association can be explained by a stabilizing effect of GRK6 on the von Hippel-Lindau tumor suppressor (pVHL), which is a main regulator of HIF1α activity [91]. Under normoxic conditions, HIF1α is hydroxylated at two proline residues (pro403 and pro564) by oxygen-dependent prolyl-4-hydroxylases, which consequently enables pVHL to bind and to direct the complex to ubiquitination and proteasomal degradation (Figure 5) [92]. Therefore, the expression of HIF-regulated genes is inhibited. Under hypoxic conditions, owing to the lack of hydroxylase-substrates, the level of non-hydroxylated HIF1α rises, which translocates to the nucleus and forms a dimer with HIF1β (Figure 5) [92]. Hereinafter, the complex binds hypoxia response elements (HRE) and further induces transcription [93]. In inflammation, HIF1α contributes to maturation of dendritic cells, expression of lymphangiogenic cytokines, as well as T-cell activation, is linked to Toll-like receptor 4 and signal introducer and activator of transcription 3 (STAT3) signaling, and may be an important mediator of NFκB-mediated transcription under hypoxic conditions (reviewed in [94]). By supporting pVHL stability, GRK6 may therefore contribute to the suppression of HRE-mediated genes. The precise underlying mechanism, however, still has to be elucidated.

Furthermore, GRK6 expression is negatively correlated with platelet-derived growth factor β receptor (PDGF-R) signaling, and in addition, activation of PDGF-R resulted in Src-mediated proteasomal degradation of GRK6 protein (Table 1) [86]. Finally, GRK6 was described to phosphorylate STAT3 [7].

## 9. GRK6 as a Potential Therapeutic Target in Treating Chronic Inflammatory Diseases

In the challenge of identifying possible therapeutic targets for the treatment of chronic inflammatory diseases as well as for acute inflammatory responses, GRK6 may be a promising candidate. Its downregulation seems to be closely related to the development of chronic inflammatory diseases and goes along with a disbalance of multiple pathways regulating and maintaining pro-inflammatory cell reactions. For example, in GRK6 deficiency, TNF-α and IL-1β signaling seems to be disrupted, and the expression of NFκB-regulated cytokines might be impaired [49,87]. In the context of chronic inflammatory diseases, drug-induced upregulation of GRK6 expression could alleviate post-inflammatory hyperalgesia and abrogate prolonged cellular inflammatory response. Conversely, GRK6 inhibition could be a potential therapeutic approach to treat osteoarthritis, which is associated with an upregulation of GRK6 [90].

Since GRK6-knockout mice are fully viable and show no obvious disabilities under basal conditions, inhibition of GRK6 may be tolerable in vivo. In vitro, GRK6 knockdown led to an increased rate of apoptosis of 10% in human epithelial cells, which was considered to be acceptable [7]. A specific evaluation of vulnerability of other tissues, nevertheless, is still pending.

Elevating GRK6 expression levels could be realized on a transcriptional level, for instance by using identified transcription factor binding sites, such as CREB or C/EBPα [36,37]. Since both transcription factors are, however, neither specific to GRK6 gene regulation, nor specifically expressed in certain tissues or cell types, an alteration of their transcriptional activity would possibly have devastating effects on the cellular maintenance of non-participating organs or cellular systems. Other starting points to target GRK6 may be located on a post-transcriptional or post-translational level. Mainly GRK6 selective inhibition or upregulation could be realized by taking advantage of micro-RNAs (miR). Since GRK6 was identified as a direct target of miR-19b-3p, it could represent a potential target of altering intracellular GRK6 expression levels post-transcriptionally [90]. Micro-RNAs (miR) play a decisive part in the post-transcriptional regulation of inflammatory cascades, for instance by abrogating TNF-α, macrophage chemoattractant protein-1 or cyclooxygenase-2 expression [95]. In osteoarthritis, miR-19b-3p was shown to be downregulated, whereas GRK6 expression levels were elevated [90]. Experimental suppression of GRK6 expression led to a decrease in IL-8 production and an increase in the expression of proteins of the extracellular matrix, and this process could be induced by treatment with miR-19b-3p [90].

Additionally, GRK6 is bound by heat shock protein 90 (HSP90), and its inhibition led to a distinct decline in GRK6 expression in myeloma cells [7]. Therefore, the inhibition of heat shock proteins might also be a post-translational approach to GRK6 downregulation in a broader range of tissues, which will require further investigations.

Another opportunity to therapeutically address GRK6 expression is for instance the use of known pathways leading to GRK6 downregulation. Proteasomal degradation of GRK6 might be related to PDGFR-signaling [86]. Albeit, in this context, exact mechanisms leading to degradation are not resolved yet and require further investigations.

An intriguing approach to treatment in chronic inflammation might also be the specifically targeted inhibition of signal transduction directly at the receptor, for instance by using the COMMD3/8 complex to silence GRK6-mediated downstream cascades [25]. Since these findings are still quite new, the interactions of COMMD3/8 and GRK6 will need to be further investigated in detail.

In contrast to GRK2 [96], selective drug-like inhibitors or activators of GRK6 activity in vivo are not identified so far. Due to its upregulation in various malign tumour tissues, however, there are several promising efforts to identify specific GRK6 inhibitors in oncological settings. Notably, experimental in vitro inhibition of Src using dasatinib resulted in increased GRK6 expression in a medulloblastoma cell model [86]. Furthermore, GRK6 was successfully inhibited by treatment with GF109206X in vitro, alongside to an inhibition of protein kinase C, leading to cytotoxicity in myeloma cell lines [7]. The same group identified a selective GRK6 inhibitor based on 4-aminoquinazoline acting synergistically to bortezomib, which is a well-established treatment for multiple myeloma [97].

Unfortunately, GRK6 expression as well as its exertion of influence on cellular processes and downstream signaling pathways seems to be cell type specific and, in some cases elucidated so far, contradictory. For example, its response to CXCL12 stimulation concerning chemotaxis and leukocyte diapedesis is not resolved in full extension [67,68].

Furthermore, GRK6 may only represent one of an abundance of possible targets in the treatment of inflammation. A combined therapy, also including other GRK subtypes, is therefore conceivable.

So far, studies showed that GRK6 is part of a large and hitherto not fully understood interactome. The entire range of possible interactions is still unclear, but its understanding is a prerequisite to develop a realistic therapeutic approach allowing a reasonable appraisal of adverse effects. Therefore, further investigations will have to shed light on the precise regulatory mechanisms behind the alteration of GRK6 expression under inflammatory circumstances in the future.

## 10. Conclusions

GRK6 is a crucial regulator of inflammation-related signaling pathways. Its interactome reaches far beyond the traditional concept of homologous receptor desensitization.

Since GRK6 is highly expressed in lymphatic tissue, it was identified as an important regulator of chemokine receptor signaling and plays a key role in directing chemotaxis and leukocyte extravasation.

In clinical and experimental settings, GRK6 downregulation is associated with chronic inflammatory diseases such as rheumatoid arthritis, chronic inflammatory bowel disease and severe inflammatory response syndrome. Decreased GRK6 expression levels may contribute to autoimmunity by reduced engulfment of apoptotic material. Furthermore, GRK6 is pivotal in mediating neuroinflammation, which was shown in different studies using a model of chronic nerve constriction injury.

GRK6 has a pivotal impact on transcriptional activity in immune cells—for example, by promoting NFκB-mediated transcription, or by repressing HIF1α-dependent transcription.

Nevertheless, the exact role of GRK6 in mediating inflammatory responses seems to be cell type specific and is not fully understood yet. Factors leading to GRK6 up- or downregulation, and downstream signaling pathways preferentially chosen under specific inflammatory circumstances, still pose a conundrum and need to be further investigated.

GRK6 represents a promising target for future therapeutical approaches to treatment of chronic inflammatory diseases as well as of acute inflammatory exacerbation.

## Data Availability

Not applicable.
